# Acute aerobic exercise effects on cognitive function in breast cancer survivors: a randomized crossover trial

**DOI:** 10.1186/s12885-019-5589-1

**Published:** 2019-04-23

**Authors:** Elizabeth A. Salerno, Kendrith Rowland, Arthur F. Kramer, Edward McAuley

**Affiliations:** 1 Cancer Prevention Fellowship Program, Division of Cancer Epidemiology & Genetics, Metabolic Epidemiology Branch, National Cancer Institute, Bethesda, MD 20892 USA; 2 University of Illinois at Urbana-Champaign, Champaign, USA; 3 Carle Foundation Hospital, Urbana, USA; 4 Northeastern University, Boston, USA

**Keywords:** Breast cancer, Acute exercise, Cognitive function, Survivorship

## Abstract

**Background:**

Many breast cancer survivors (BCS) report deficits in cognitive function. Physical activity (PA) has been associated with better processing speed and memory in healthy adults and thus may be a useful method for improving cognition in BCS. The purpose of this study was to examine the effects of an acute bout of PA on processing speed and spatial working memory in a sample of BCS.

**Methods:**

Using a repeated measures, crossover design, BCS [*N* = 27; *M*_age_ (SD) = 49.11(8.05)] completed two sessions in counterbalanced order: 30 min of moderate-intensity treadmill walking and 30 min of seated rest. Women completed cognitive tasks immediately before and after each session.

**Results:**

Within-subjects repeated measures analyses of variance revealed a significant time by session effect for processing speed reaction time [F (1,25) = 5.02, *p* = .03, η2 = 0.17]. This interaction was driven by significantly reduced reaction time (e.g., faster response) post-exercise and no change post-rest. Further between-subjects analyses indicated a significant time by session by moderate to vigorous physical activity (MVPA) split [F (1,25) = 5.23, *p* = .03, η2 = 0.17], such that women who engaged in ≥45 min of average daily MVPA reduced their reaction time post-exercise (*p* = .01) and increased RT post-rest (*p* = .06). Time by session effects for spatial working memory 3-item accuracy and 4-item reaction time trended towards significance, *p* = 0.08 and *p* = 0.10, respectively, again driven by better performance post-exercise.

**Conclusions:**

The moderate effect of acute exercise on domains of memory and processing speed in BCS is encouraging. Cancer-related cognitive impairment remains largely misunderstood; however, the results from the present study offer preliminary evidence for the positive relationship between acute exercise and cognition in BCS.

**Trial registration:**

ClinicalTrials.gov NCT02592070. Registered 30 October 2015. Retroactively registered.

## Background

Breast cancer mortality in the United States has been declining over the past two decades [1, 2]; however, the incidence of breast cancer is steadily rising with an estimated 1 in 8 women expected to develop the disease over the course of her lifetime [1]. Consequently, the number of women living with a history of breast cancer is expected to reach 4 million by the year 2020 [3]. While effective in improving survival rates [4, 5], cancer treatment is associated with a host of deleterious health consequences, ranging from reduced physical function to increased risk of developing comorbid diseases [6, 7]. One such detriment is impaired cognitive function, highlighted by the National Coalition for Cancer Survivorship as a significant quality of life concern for cancer survivors [8].

Cancer-related cognitive impairment (CRCI) has been defined in the literature as the loss of mental acuity associated with cancer and its subsequent treatment [9]. Working memory, the act of holding information in one’s mind and manipulating it, and processing speed, the speed with which one interprets information [10], have both been identified as important determinants of long-term survival in cancer survivors [11–13]. Unfortunately, these are the same cognitive processes most commonly reported by survivors to be negatively affected throughout their cancer experience [14]. Janelsins and colleagues [15] reported that CRCI can be detected in up to 30% of patients prior to treatment, 75% during treatment, and 35% up to a decade post-treatment. The etiology of CRCI in breast cancer remains unclear, but is hypothesized to be multi-factorial with influence from genetic, psychosocial, treatment-specific (e.g., chemotherapy), and behavioral factors, among others [15, 16]. It is also theorized that cancer and its treatment accelerate the aging process, initiating cognitive decline sooner in survivors than the general population [17]. As such, there’s a burgeoning need to identify effective and low-cost methods for improving cognitive and brain health in this cancer cohort to ultimately improve quality of life during survivorship.

Physical activity (PA) is a lifestyle behavior that has been consistently associated with improved physical, cognitive and mental health across the lifespan [18]. Recent work has attempted to understand the effects of chronic exercise training on cognition in cancer survivors with mixed, albeit tentatively promising, results [19–21]. Unfortunately, breast cancer survivors generally fail to meet federal recommendations for PA [22]. Given the many difficulties survivors face in initiating and maintaining an exercise regimen (e.g., fatigue, pain), acute PA, or single exercise sessions, may be a more achievable and salient target for a subset of this cohort, particularly if immediate health improvements can be evidenced.

The recent release of the 2nd edition of the Physical Activity Guidelines for Americans in 2018 has highlighted strong evidence for acute PA in improving cognition across the lifespan [23]. In healthy adults, a meta-analysis by McMorris and colleagues [24] found significant improvements in reaction time on working memory tasks following acute bouts of moderate-intensity PA. Similarly, another meta-analysis [25] in disease-free individuals found a small, positive effect of acute aerobic exercise on varying domains of cognition across the lifespan. In breast cancer survivors specifically, several studies have indicated that an acute bout of exercise may improve psychological and physical health components [26–28]. Despite the documented evidence of acute exercise benefits, to our knowledge, no study to date has examined the cognitive effects of acute exercise in breast cancer survivors specifically.

The primary aim of the present study was to examine the effects of a 30-min moderate-intensity aerobic exercise session on processing speed and spatial working memory compared with 30 min of quiet, seated rest in breast cancer survivors. Because of previous work demonstrating acute exercise benefits in both non-diseased adults and breast cancer survivors [17, 18, 21, 22], we hypothesized that participants would improve (e.g., greater accuracy, faster response time) in domains of processing speed and spatial working memory after exercise compared with after rest. We also sought to examine how regular PA may have influenced the relationship between acute exercise and cognitive function. Due to the heavily documented positive effects of chronic exercise on cognition across the lifespan [18], we hypothesized that women who engaged in higher levels of regular PA would exhibit greater performance compared with their less active counterparts.

## Methods

### Participants

**Fig. 1:**
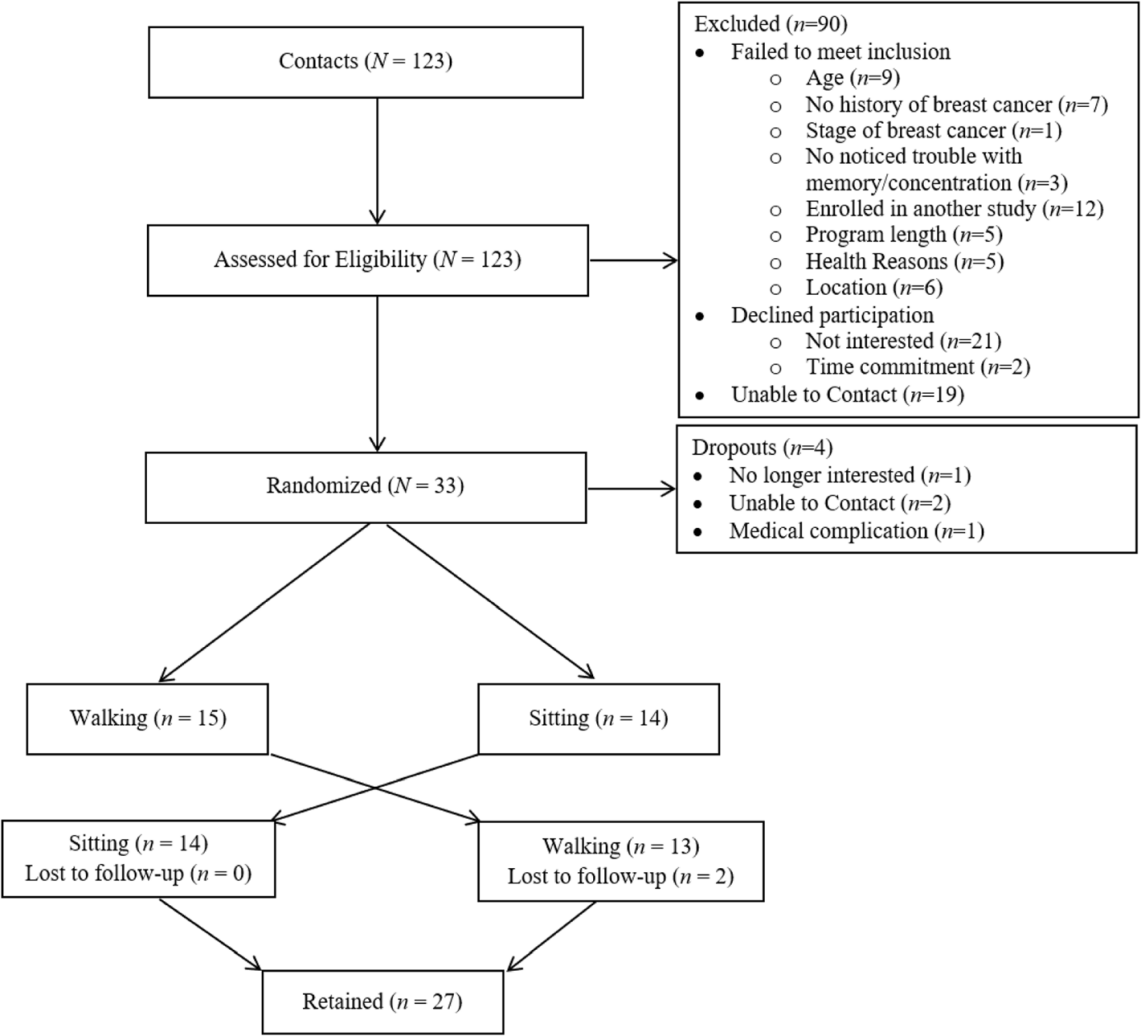
CONSORT. Detailed flow of participants through the study

Community-dwelling breast cancer survivors were recruited to participate in a randomized, crossover study assessing the effects of acute exercise on cognitive functioning. Recruitment efforts included local media, the local university e-newsletter, and family, friend, and Carle Foundation Hospital oncologist referral. Eligibility criteria required participants to be: female; between the ages of 30 and 60; a physician-confirmed breast cancer survivor (stages DCIS-IIIB); completed with primary treatment for breast cancer; capable of participation in maximal exercise as determined by their personal physician; free of dementia or organic brain syndrome; capable of walking unassisted; suffering from self-reported memory troubles after diagnosis/treatment; and free from other health reasons contraindicating exercise. Participant flow through the study is detailed in Fig. 1. The trial ran from October 2015 to April 2016, the scheduled date of closure. All methods and procedures were approved by the institutional review board (IRB; ethics committee) at the University of Illinois at Urbana-Champaign, conducted in Urbana, IL, and written informed consent was obtained from all individual participants included in the study.

### Procedures

Participants completed three appointments. The first consisted of a graded maximal exercise test to determine maximal heart rate. At the second and third appointments, participants completed 30 min of either exercise or rest with pre- and post- cognitive function measures. These two sessions were counterbalanced using block randomization (1:1 allocation ratio; block size of 4) after passed prescreening to control for potential learning and/or practice effects on the cognitive tasks. The primary study investigator generated the random allocation sequence, and the first author enrolled and assigned participants to interventions.

### Aerobic exercise session

The aerobic session consisted of two rounds of cognitive tasks as well as a 30-min bout of moderate-intensity aerobic exercise. Upon arrival, participants completed the battery of cognitive tasks (detailed later) in a quiet, distraction-free room. Upon completion of the cognitive battery, individuals were fitted with a heart rate monitor and briefed on the aerobic walking session. Participants were instructed to walk on a treadmill at 40–60% of their maximal heart rate (HR) as determined by their individual graded exercise test. Treadmill speed and/or elevation were defined through a cooperative effort from both the participant and trained exercise specialist to ensure the appropriate HR range as well as participant comfort and safety. To account for varying heart rates that may have prevented participants from achieving the desired range (e.g., use of beta blocker medications), participants also maintained a rating of perceived exertion (RPE) between 8 and 11 throughout the 30 min [29]. This session began and ended with a 2-min warm-up and cool-down period, respectively. To prevent the potential confounding influence of social interaction between the participant and exercise leader, talking was kept to a minimum. HR, RPE, and blood pressure (BP) were measured before, during, and after the session as needed for safety. Upon completion, participants removed the heart rate monitor and repeated the cognitive battery.

### Seated rest session

The seated resting session consisted of two rounds of the cognitive tasks as well as a 30-min period of seated rest. Upon arrival, participants completed the cognitive battery in the same quiet, distraction-free room. Once completed, participants were fitted with a heart rate monitor and instructed to remain seated for 30 min with the option to watch a television show. They were told to refrain from talking, reading, using their phones or falling asleep. HR, RPE, and BP were assessed before, during, and after the session. Upon completion, participants completed their second cognitive battery.

### Measures

#### Demographics & health history

Participants self-reported age, race, education, income, education and breast cancer diagnostic history. Body mass index (BMI) was assessed via height and weight measurements taken on a calibrated stadiometer at the first appointment.

#### Cognitive battery

The cognitive battery was delivered immediately before and after each exercise and resting session and was comprised of the following tasks: processing speed and spatial working memory (described in detail below). All task instructions were presented for participants to read followed by a practice round. Response accuracy feedback was given during practice rounds only. A trained staff member was present in the room for all practices to answer any questions and troubleshoot comprehension issues. The staff member then exited the room before the trial began.

#### Letter comparison task

The letter comparison task was used to measure processing speed. This paper and pencil task consisted of two pages containing strings of consonants separated by a line (ex: TGL ___ YGL) [30]. Participants were asked to classify the pairs as either “same” or “different” by writing an “S” or “D” on the line as appropriate. They were encouraged to work in order from the top of the page to the bottom as quickly and accurately as possible. Each page was timed separately. Outcome variables from this test were accuracy (e.g., percentage of correct responses) and reaction time (e.g., length of time taken to complete the task).

#### Spatial working memory task

The spatial working memory task was a computer-based cognitive task requiring participants to focus on a cross in the middle of a white screen. Two, 3, or 4 black dots appeared on the screen for a duration of 500 milliseconds (ms) before disappearing. A red dot then appeared for 2000 ms [31]. Participants were asked to indicate whether the location of the red dot matched one of the previous black dot locations using keys on a keyboard (“M” for *match* or “X” for *no match*) as quickly and accurately as possible. Outcome variables were accuracy (e.g., percentage of correct responses) and reaction time (e.g., length of time taken to respond) for each of the trials (2-item: 2 dots; 3-item: 3 dots; 4-item: 4 dots).

#### Physical activity

PA was assessed objectively via accelerometry using Actigraph brand accelerometers (Actigraph, Pensacola, FL: model GT3X). Each participant was instructed to wear her accelerometer for seven consecutive days on her non-dominant hip during all waking hours and record the time worn on a log sheet. Data were scored with an interruption period of 60 min, and all data retained for analyses met at least 10 h of wear time on at least 3 days [32]. Accelerometry data were then downloaded as activity counts representing raw accelerations summed over a 1-s epoch length that varied based on intensity and frequency of the accelerations [33]. All downloaded data were then analyzed in ActiLife (Version 6; Actigraph, Pensacola, FL) using adult-specific intensity (counts/minute) cut-points [34]. To determine average daily moderate-to-vigorous physical activity (MVPA), the number of minutes spent engaging in MVPA was divided by the total number of valid days worn per participant. For the analyses reported herein, we used a median split of average daily MVPA (e.g., < 45 min and ≥ 45 min) to identify differences in cognitive outcomes between regular exercisers and their less active counterparts.

### Data analysis

Twenty-four participants provided 80% power assuming a 0.05 alpha to detect a moderately-sized effect (i.e., *η*^2^ ≥ .07) from pre- to post-session between exercise and rest. Cohen’s D effect sizes were also calculated. Defined as the standardized difference between treatment and comparison group means [35], effect sizes are independent of sample size and thus may be better indicators of group differences than traditional *p* values [36]. It remains unclear if such a change in cognitive reaction time and accuracy is clinically meaningful in breast cancer survivors; however, there are currently no studies examining the effects of acute aerobic exercise on cognitive function in this cancer cohort. Findings from this study will provide effect sizes for future work replicating these efforts.

All analyses were conducted in SPSS (Version 22; Chicago, IL). Initial analyses used two (session) by two (time) repeated measures analyses of variance to examine the effects of the two sessions on processing speed and spatial working memory. Given that the design of this study exposed participants to both the exercise and resting sessions, time (e.g., pre- and post-session) and session (e.g., exercise and rest) were included as within-subjects factors to allow each subject to act as her own “control”. We then used the average daily MVPA median split as a between-subjects factor to examine the differential effects of acute exercise on cognition by regular MVPA status (e.g., ≥45 min MVPA/day vs. < 45 min MVPA/day). Scores below 50% on accuracy outcomes were removed and considered missing. Such scores are worse than chance making it likely that participants either did not comprehend the task or were guessing, resulting in an unreliable score. As such, if a participant’s score for accuracy was below this threshold for one session, her other session was marked missing as well. Final sample sizes for each cognitive outcome are detailed in Figure legends.

## Results

A total of 27 women completed all testing, *M*_age_ = 49.11(8.05). Table 1 depicts sample and cancer-specific characteristics.

**Table 1:** Sample Characteristics

	Mean (SD) or % *N* = 27
Age	49.11 (8.05)
Cancer Stage	
DCIS	7.1%
I	39.3%
II	35.7%
III	17.9%
Estrogen receptor positive	71.4%
Treatment	
Chemotherapy	82.1%
Months since chemotherapy	54.7 (60.3)
Radiation therapy	75.0%
Months since radiation therapy	64.1 (66.0)
Surgery	100%
Months since surgery	53.2 (45.4)
Marital Status	
Married	70.4%
Employment Status	
Full Time (> 35 h/wk)	74.1%
Race	
White	100%
Highest Level of Education	
>College Degree	66.6%
Annual Household Income	
<$45,000	18.5%
≤$90,000	14.8%
>$90,000	37.0%
Chose not to answer	29.6%
Body Mass Index (BMI)	
Normal Weight	34.6%
Overweight	30.8%
Obese	34.6%

*SD* standard deviation, *DCIS* ductal carcinoma in situ

Briefly, participants were stage I or II survivors (75%), recipients of chemotherapy (82.1%), married (70.4%), employed full-time (74.1%), college educated (66.6%), and Caucasian (100%). Mean data (e.g., HR, BP, RPE) from the exercise and resting sessions are detailed in Table 2. No adverse events occurred through the duration of this project.

**Table 2:** Mean Data from Aerobic & Resting Sessions

	Exercise Session Mean (SD) *N* = 27	Resting Session Mean (SD) *N* = 27
Heart Rate (bpm)	103.70 (12.15)	74.33 (13.09)
Systolic Blood Pressure (mmHg)	123.77 (13.20)	108.00 (11.13)
Diastolic Blood Pressure (mmHg)	74.15 (7.37)	71.00 (8.04)
Rating of Perceived Exertion	8.74 (1.93)	6.11 (0.32)
Time Between Session and Cognitive Tasks (min)	5.07 (1.38)	2.28 (1.10)

*SD* standard deviation, *bpm* beats per minute, *mmHg* millimeter of mercury

### Processing speed

**Fig. 2:**
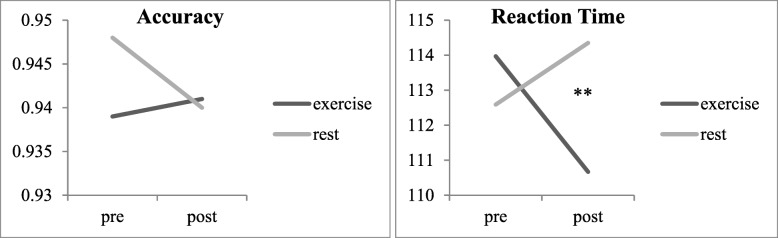
Processing speed changes across time and session. Accuracy *n* = 27; Reaction Time *n* = 26. ** significant at *p* < .05

**Fig. 3:**
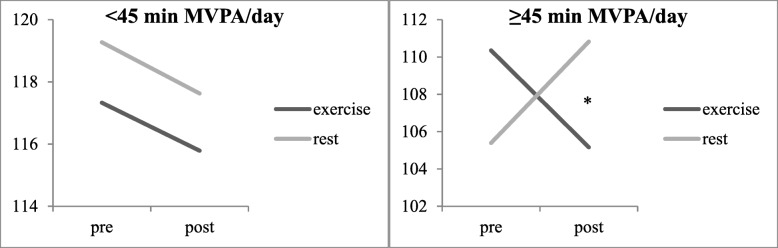
Processing speed reaction time changes across time, session, and MVPA split. Accuracy *n* = 27; Reaction Time *n* = 26. * significant at *p* = .01

Our initial analyses comparing the differential effects of exercise and rest on processing speed revealed a significant time by session interaction for reaction time [F (1,25) = 5.02, *p* = .03, η^2^ = 0.17], such that participants were significantly faster from pre- to post-exercise (*p* = .02) compared with no change from pre- to post-rest (p = .33) as shown in Fig. 2 (*d* = .25). Further between-subjects analyses indicated a significant time by session by MVPA interaction [F (1,25) = 5.23, *p* = .03, η2 = .17]. This was driven by more significantly faster reaction time from pre- to post-exercise (*p* = .01) in women with at least 45 min of MVPA/day (*n* = 13) compared with slower reaction time from pre- to post-rest (*p* = .06) as shown in Fig. 3 (*d* = .65). There were no significant changes in reaction time after either session (e.g., exercise, rest) for women engaging in less than 45 min of MVPA per day (*n* = 14). There was no significant time by session interaction for accuracy (*p* = .44), thus between-subjects analyses for MVPA were not explored.

### Spatial working memory

**Fig. 4:**
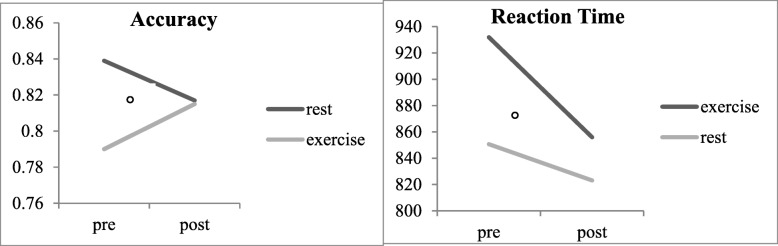
Spatial working memory changes across time and session. 3-item Accuracy *n* = 27; 4-item Reaction Time *n* = 24. **°** trend at .08 *< p <* .10.

Analyses comparing the differential effects of exercise and rest on spatial working memory revealed a time by session interaction that trended towards significance for 3-item response accuracy [F (1,26) = 3.36, *p* = .08, η^2^ = 0.12]. This effect was explained by increased accuracy post-exercise compared with reduced accuracy post-resting session (*d* = 0.48) as shown in Fig. 4. The time by session interaction for 4-item reaction time was also nonsignificant [F (1,23) = 2.93, *p* = .10, η^2^ = 0.11], but trended towards reduced reaction time post-exercise session compared with no change in reaction time post-resting session (*d* = 0.29) as shown in Fig. 4. Further between-subjects analyses for MVPA were not significant (*p* = .33).

## Discussion

The purpose of this study was to examine whether acute bouts of exercise and rest differentially influenced processing speed and spatial working memory in breast cancer survivors. Although there is evidence for the use of exercise in improving varying domains of physical and mental health in breast cancer survivors [26, 27], this study is the first, to our knowledge, to examine the immediate effects of moderate-intensity exercise on domains of cognitive functioning in breast cancer. In the present study, there was a significant time by session interaction for processing speed reaction time and a time by session interaction that trended towards significance for spatial working memory accuracy. Notably, the effect sizes for these interactions ranged from small (*d* = .25) to moderate (*d* = .65) in favor of exercise, suggesting that the results reported herein are indeed meaningful for future work. Importantly, these findings are within the context of a single walking session: an achievable method with the capacity for real world translation beyond a cancer diagnosis. However, we acknowledge that this is nascent research in a small sample and further work exploring acute exercise effects on cognition after cancer is warranted.

Our analyses examining differential effects of exercise and rest on processing speed demonstrated a significant time by session effect for reaction time such that women displayed reduced reaction time (e.g., performed faster) after exercise compared with no change in reaction time after rest. Response accuracy after each session remained unchanged (*p*s > .38), suggesting that women performed faster after exercise without sacrificing accuracy. Given that there are no comparable studies in the literature examining the effects of acute exercise of cognition in breast cancer survivors with which to make comparisons, we discuss the current results relative to previous research in healthy adults. Indeed, these findings are consistent with the acute exercise literature such that individuals benefit from acute exercise in significantly improved reaction time but not response accuracy [24, 37]. McMorris and colleagues [38] have previously discussed the inherent discrepancy between the cognitive nature of the task and the motoric response required, possibly explaining the differential effects of acute exercise on speed and accuracy. After replicating these findings on a larger scale, further work examining the underlying biological pathways contributing to these differential effects of exercise on cognitive outcome variables in breast cancer survivors is necessary.

Of further interest is the moderating effect of chronic MVPA on the relationship between acute exercise and processing speed. In the current study, women who accumulated at least 45 min of MVPA per day on average performed significantly faster on the processing speed task after exercise compared with their less active counterparts (*d* = .65). The moderate magnitude of this effect suggests that the influence of chronic MVPA on the relationship between acute exercise and cognition in breast cancer is meaningful. This finding supports the well-documented relationship between regular PA engagement and improved cognition in healthy individuals across the lifespan [39–41], suggesting that breast cancer survivors may be amenable to similar cognitive benefits through exercise. It is also possible that 30 min was too long an exercise duration for women who were not regular exercisers, despite its moderate intensity. While all acute sessions were individualized to the women’s fitness levels, fatigue may have been a contributing factor for those who were deconditioned and/or more sedentary [25]. Future research might explore potential dose-response effects of exercise duration and/or intensity on cognition to determine the optimal length of exercise in this cancer cohort.

Interestingly, while women who accumulated at least 45 min of MVPA/day had faster reaction after walking for 30 min, their performance trended slower after sitting. This may be due to the control condition (television viewing) of the present study. Indeed, there is a growing body of literature highlighting the negative influence of television viewing/screen time on varying domains of cognition across the lifespan [37, 42, 43]. While this context is likely a good proxy for real-world sedentary behavior, it will be important for future research to include other control conditions (e.g., reading, socializing) to further tease out the effects of acute exercise on cognition after cancer.

In addition to improving processing speed, women in the present study marginally improved response accuracy on a spatial working memory task immediately after acute exercise. This finding contrasts previous work in healthy adults demonstrating small to moderate decrements in accuracy post-exercise [24]. It may be that breast cancer survivors have more room for accuracy improvements in certain cognitive domains after acute exercise, particularly if they are starting at lower levels of cognition prior to lifestyle intervention. Notably, participants in the current study were eligible if they self-reported noted trouble with memory or concentration after their cancer experience, therefore they may have had more room for improvement than the population level of breast cancer survivors. Although statistically nonsignificant (*p* = .08), the effect size for the time by session effect on spatial working memory accuracy was moderate in scale (*d* = .48). Effect sizes are independent of sample size and thus are considered better indicators of group differences than traditional *p* values [36]. In this case, the magnitude of the effect suggests that exercise may have a meaningful influence on spatial working memory, an important implication for future intervention designs targeting cognitive health in breast cancer survivors.

These findings are timely given the burgeoning interest in acute exercise on a national level [23]. With a growing body of literature suggesting beneficial effects of chronic exercise on cognition in healthy individuals, it will be important to consider the role of acute exercise in this relationship. There is evidence for differential effects of acute and chronic exercise on cognitive and affective outcomes [44], which is critical for informing behavioral change interventions. It’s important that future work disentangle their confluence, specifically within the context of cancer and its heterogeneous manifestation. While we present nascent work in a small sample of breast cancer survivors, these findings suggest that acute exercise may be a complementary area of research worth exploring in tandem with long-term PA for sustained cognitive health and quality of life during survivorship.

There are several limitations to the present study. The sample size was small and comprised of white, highly educated women, therefore findings are certainly not generalizable across all breast cancer survivors. Given cancer’s wide-ranging presentation and consequential disparities that exist across the continuum, future work should target a larger, more demographically diverse sample. In addition, these results are specific to post-treatment survivors. Knowing that CRCI exists across the entirety of the cancer continuum, it will be important to determine how acute exercise may be leveraged for cognitive health both prior to and during treatment as well as within different subgroups of cancer and treatment regimens. It is possible that the findings herein may be magnified in women undergoing treatment with higher levels of CRCI [15]. However, it is nevertheless exciting that acute exercise has the capacity to significantly improve cognition in a sample of breast cancer survivors that is, on average, ~ 4.5 years post-treatment. Similarly, these findings are relevant to only two domains of cognitive function. Understanding the effects of acute exercise on other domains of executive functioning such as attention or cognitive flexibility will be important. More research is warranted to replicate and extend the findings reported herein to provide greater insight into the relationship between acute exercise and cognition in cancer survivors.

## Conclusions

Despite these limitations, 30 min of walking may be a better choice than sitting for at least maintaining, if not improving, important domains of cognitive functioning in breast cancer survivors. As the population continues to age and more individuals live beyond their cancer diagnosis, it will become increasingly important to understand and prevent its deleterious health effects. Cancer-related cognitive impairment remains largely misunderstood; however, results from the present study offer preliminary evidence for the positive association between acute exercise and cognitive function in breast cancer survivors.

## Abbreviations

BCS: Breast cancer survivors
BMI: Body mass index
BP: Blood pressure
DCIS: Ductal carcinoma in situ
HR: Heart rate
IRB: Institutional review board
ms: milliseconds
MVPA: Moderate-vigorous physical activity
PA: Physical activity
RPE: Rating of perceived exertion
SD: Standard deviation
